# Analysis and Validation of Hub Genes in Blood Monocytes of Postmenopausal Osteoporosis Patients

**DOI:** 10.3389/fendo.2021.815245

**Published:** 2022-01-13

**Authors:** Yi-Xuan Deng, Wen-Ge He, Hai-Jun Cai, Jin-Hai Jiang, Yuan-Yuan Yang, Yan-Rong Dan, Hong-Hong Luo, Yu Du, Liang Chen, Bai-Cheng He

**Affiliations:** ^1^ Department of Pharmacology, School of Pharmacy, Chongqing Medical University, Chongqing, China; ^2^ Key Laboratory of Biochemistry and Molecular Pharmacology of Chongqing, Chongqing Medical University, Chongqing, China; ^3^ Department of Orthopaedics, The Second Affiliated Hospital, Chongqing Medical University, Chongqing, China; ^4^ Department of Bone and Soft Tissue Oncology, Chongqing University Cancer Hospital, Chongqing, China

**Keywords:** postmenopausal osteoporosis, microarray, enrichment analysis, hub genes, OVX

## Abstract

Osteoporosis is a common systemic bone disease caused by the imbalance between osteogenic activity and osteoclastic activity. Aged women are at higher risk of osteoporosis, partly because of estrogen deficiency. However, the underlying mechanism of how estrogen deficiency affects osteoclast activity has not yet been well elucidated. In this study, GSE2208 and GSE56815 datasets were downloaded from GEO database with 25 PreH BMD women and 25 PostL BMD women in total. The RRA algorithm determined 38 downregulated DEGs and 30 upregulated DEGs. Through GO analysis, we found that downregulated DEGs were mainly enriched in myeloid cell differentiation, cytokine-related functions while upregulated DEGs enriched in immune-related biological processes; pathways like Notch signaling and MAPK activation were found in KEGG/Rectome pathway database; a PPI network which contains 66 nodes and 91 edges was constructed and three Modules were obtained by Mcode; Correlation analysis helped us to find highly correlated genes in each module. Moreover, three hub genes FOS, PTPN6, and CTSD were captured by Cytohubba. Finally, the hub genes were further confirmed in blood monocytes of ovariectomy (OVX) rats by real-time PCR assay. In conclusion, the integrative bioinformatics analysis and real-time PCR analysis were utilized to offer fresh light into the role of monocytes in premenopausal osteoporosis and identified FOS, PTPN6, and CTSD as potential biomarkers for postmenopausal osteoporosis.

## Introduction

Osteoporosis is a common systemic bone disease caused by the decrease of bone density and bone mass, the destruction of bone microstructure, the increase of bone fragility and the easy occurrence of fracture due to a variety of reasons (1). One in three women and one in five men over the age of 50 are at risk of osteoporotic fractures. The prevalence of osteoporosis in people over 50 years of age was 19.2%, 6.0% for men and 32.1% for women in China. The prevalence of osteoporosis in people over 65 years of age was as high as 32.0%, with 10.7% in men and 51.6% in women (2). According to the pathogenetic factors of osteoporosis, it can be divided into primary osteoporosis and secondary osteoporosis. The former includes postmenopausal osteoporosis and senile osteoporosis, of which postmenopausal osteoporosis is one of the most common types of osteoporosis (3).

Postmenopausal osteoporosis (PMOP) is a metabolic disease that is caused by the reduction of ovarian function and decreasing estrogen levels in postmenopausal women (4). The etiology of PMOP is complex, and the precise mechanism is yet unknown. A variety of signaling pathways and immune factors, such as receptor activator of nuclear factor-κB/receptor activator of nuclear factor-κB ligand/osteoprotegerin, Wnt/β-catenin, Semaphorin3A/neuropilin-1, peroxisome proliferator-activated receptor, and others, may be involved in the regulation of PMOP, which form a regulatory network in the body and cause an imbalance in the process of bone remodeling, and when the bone destruction is greater than the bone formation, PMOP ultimately manifests (5–8).

It is well known that osteoporosis is closely related to bone formation, remodeling, and resorption, which mainly depends on the activity of osteoblasts and osteoclasts (9). Osteoblasts are the major cellular components of bone, derived from mesenchymal stem cells, and abundant in periosteum, the thin connective tissue layer on the outer surface of bone and the intima (10). They deposit new bone minerals through a mechanism that has not yet been fully described. Osteoclasts are multinucleated cells derived from bone marrow hematopoietic progenitor cells. They have highly active ion channels on the membrane and can pump protons into the extracellular space, thereby reducing the pH of their own microenvironment to promote bone resorption (11). Although there has been significant progress in pathogenesis for osteoporosis, effects of blood monocytes on osteoporosis, especially postmenopausal osteoporosis, need to be further elucidated.

In recent years, with the continuous development and maturity of gene detection technology, microarray and high-throughput sequencing technology are increasingly widely used to search for potential biomarkers related to diagnosis, treatment, and prognosis. It is well known that public databases such as the Gene Expression Omnibus (GEO) are widely used to explore diagnostic and prognostic biomarkers for many diseases. Meanwhile, in order to overcome the limited or inconsistent results caused by different technology platforms and small sample sizes, robust rank aggregation (RRA) was used to obtain robust difference expressed genes (DEGs) (12). This method is widely used in the comprehensive analysis of multiple data sets and is robust to errors and noise (13–15). However, there have been no reports of RRA in postmenopausal osteoporosis.

Animal models of osteoporosis can be used to investigate new techniques of prevention and therapy of osteoporosis. The ovariectomy rat model (OVX) is the first alternative, and it is the most commonly utilized approach in such investigations (16). The ovariectomy rat model is an excellent preclinical model for postmenopausal osteoporosis, according to FDA recommendations (17). The de-ovulatory rat osteoporosis model replicates estrogen deficiency-induced bone loss and shows postmenopausal osteoporosis clinical symptoms (18). Furthermore, it was reported that six months is the optimal time to induce OVX. The success of OVX can be verified 1–3 weeks after surgery, when the normal estrous cycle is stopped, estradiol, progesterone, and uterine weight are decreased, and LH and FSH levels are increased (19). Therefore, it is a suitable tool for the OVX model to study postmenopausal osteoporosis.

In this study, the mRNA expression data of blood monocytes of PMOP from the GEO databases were analyzed by RRA, and we further explored the development of PMOP through functional enrichment and protein-protein interaction (PPI) analysis. Then, the hub genes we got were subjected to real-time PCR analysis for further validation in the OVX model. This study provides a possible basis for understanding the etiology and potential molecular events of PMOP by analyzing the differentially expressed genes.

## Materials and Methods

### Sample Collection

Samples of monocytes were isolated from whole blood of aged women which were obtained from the Gene Expression Omnibus (GEO) database (20). Searching terms as “BMD”, “Osteoporosis”, “postmenopausal”, “Gene expression”, “Microarray”, and the datasets which are according to the following criteria will be adopted: (1) at least 10 samples each are included; (2) at least 5 cases of PMOP and 5 controls are included; and (3) raw data or series matrix file is available in GEO datasets. We finally found two datasets, GSE56815 and GSE2208 (21,22), which are completely in conformity with our criteria. Five cases of PMOP and 5 controls in GSE2208 while 20 cases of PMOP and 20 controls in GSE56815 are collected from the corresponding datasets. More information is shown on **Table 1**.

**Table 1:** Information for selected microarray datasets.

Sr. No	GEO Accession Total samples	Selected samples Platform	Source tissue
	19 samples	10 samples	blood monocytes
1	GSE2208 Sample types:	GPL96 Sample types:	
	10 high BMD	5 PreH BMD	
	9 low BMD	5 postL BMD	
	80 samples	40 samples	
2	GSE56815 Sample types:	GPL96 Sample types:	blood monocytes
	40 high BMD	20 PreH BMD	
	40 low BMD	20 postL BMD	

### Datasets Analyses

The annotated R package was downloaded through Bioconductor and the microarray probes were converted to symbols in R. The Limma package of R software was used to identify the differentially expressed genes (DEGs) associated with the PreH group and the PostL group. P-Value <0.05 and |log2 fold change (FC)| >0 were regarded as the threshold to determine the DEGs (23).

### RRA Analysis

“Robust Rank Aggregation” package (24), which could effectively reduce the errors by integrating the DEGs of these two microarray datasets and correcting for multiple times, was used to screen the robust DEGs. Genes with P-Value <0.05 and |log2 fold change (FC)| >0.5 in the ranking list were selected as upregulated/downregulated DEGs respectively for further study.

### Enrichment Analysis

Gene Ontology (GO) enrichment and the Kyoto Encyclopedia of Genes and Genomes (KEGG)/Reactome Pathway Database were used to figure out the functional roles of the Robust DEGs. The GO enrichment results, namely, biological process (BP), cellular component (CC), and molecular function (MF) were performed by using R package “clusterprofiler” with the criteria of P-Value <0.05 and P-Value <0.05. Cytoscape software plug-in ClueGO was used for pathway analysis. The criterion was that a node contained at least 3 genes and the medium Kappa score was used for pathway network connectivity (P <0.05 was considered statistically significant) (25,26). GO enrichment of Robust DEGs used the cut off criteria of P-Value <0.05. The bubble color represents P-Value in enriched pathways, and the size of the bubble represents the gene number. In the pathway enrichment analysis, each node represents a pathway, the connection between the nodes reflects the correlation between the pathways, and the size of the nodes indicates the degree of gene enrichment. All nodes contain at least three genes, and each node has a P-value <0.05.

### PPI Network Construction and its Sub-Module Analysis

By uploading the whole robust DEGs to the STRING online database (27), the PPI network which contains 66 nodes and 91 edges with confidence >0.4, was established. Pulling the results into Cytoscape (28), we visualized the PPI network and screened three key modules by using MCODE plugin.

### Correlation Analysis

After finding the key modules, we used Pearson’s correlation coefficient as an analysis method to visualize statistically significant (P-Value <0.05) and highly correlated genes in the modules by correlation analysis. Also, genes that are not statistically different are indicated by a cross. In this analysis, Package “corrplot” was used to visualize the results (29,30).

### Hub Gene Identification

To obtain as much of exact hub genes, 10 different algorithms including MCC, DMNC, MNC, Degree, EPC, BottleNeck, EcCentricity, Closeness, Radiality, and Betweenness (each approach could predict the hub genes) in Cytohubba was used (31). The first ten top ranked genes of each algorithm were regarded as the hub genes of the algorithm. After calculating, we took the one that intersects most of these algorithms and finally 3 downregulated genes which appear in nine out of ten were regarded as the most likely hub genes. Our outcome was visualized by using the R package “UpSetR” and the hub genes were represented in the red bar (32).

### OVX Model Construction

Twelve healthy female Sprague Dawley (SD) rats aged 8 weeks were purchased from the Animal Experiment Center of Chongqing Medical University. SD Rats were randomly divided into two groups: the sham operation group and the ovariectomy (OVX) group, and after 1 week of maintenance in a pathogen-free facility, bilateral ovariectomy and sham operation were performed as previously reported (33,34). Twelve weeks after surgery, the rats were sacrificed and femur and blood samples were collected and stored for further testing. This experiment was approved by the institutional animal care and use committee of Chongqing Medical University (Chongqing, China).

### Microcomputed Tomography (μ-CT) Analysis of the Proximal Femur

All the femurs were dissected, cleaned, and fixed in 4% paraformaldehyde. Then, they were scanned by a microcomputed tomography 100 (μ-CT100) system (SCANCO MicroCT). Proximal femurs were scanned by using a 20 μm voxel size, 70 kV, 200 μA and 0.6° rotation steps (180° angular range). For the trabecular bone, an area of 1.5 mm thick downward from the proximal femur growth plate was evaluated by μ-CT. This area is located 1.5 mm below the growth plate. μ-CT 516.1 software was used for 3D reconstruction and quantitative analysis. After 3D reconstruction, the bone mineral density (BMD), bone volume fraction (BV/TV), trabecular thickness (Tb.th), trabecular number (TB.n), and trabecular spacing (TB.sp) were calculated or directly measured by using μCT100 system (SCANCO MicroCT).

### Quantitative Real Time PCR Assay

Monocytes were isolated from whole blood by rat peripheral blood mononuclear cell Isolation kit. Total RNA was extracted from monocytes using SteadyPure Quick RNA Extraction Kit (AG21023-S AG). Real time PCR was performed using SYBER green mixture kit with gene specific primers (**Table 2**). The RT-PCR amplification reactions were performed using QuantStudio™ 3(Thermo Fisher SCIENTIFIC). The RT-PCR program was programmed to perform 42 cycles in total. The mean value and standard error of three independent experiments were calculated, and each experiment was repeated 3 times. The relative mRNA expression level was calculated using β-actin as internal reference.

**Table 2:** The primers used for PCR.

Gene	Primer	Sequence (5’-3’)
β-actin	F	GGAGATTACTGCCCTGGCTCCTA
	R	GACTCATCGTACTCCTGCTTGCTG
FOS	F	TGCAGACCGAGATTGCCAAT
	R	CCTCAGACTCTGGGGTGGTA
CTSD	F	GGTCCCCTCCATTCATTGCA
	R	ATGGAACCGACACAGTGTCC
PTPN6	F	TGCAGGGACGTGACAGTAAC
	R	TGACACGAGTGTTCTCCTGC

F, Forward; R, Reverse.

### Statistical Analysis

The quantitative data was presented as the mean ± SD. The two-tailed Student’s t-test was used to analyze the differences between the two groups. Each assay condition was performed in triplicate for all quantitative assays. All data collected were statistically analyzed. A P-value of less than 0.05 was defined as statistically significant.

## Results

### Identification of Robust DEGs Between Premenopausal High BMD and Postmenopausal Low BMD

**Figure 1:**
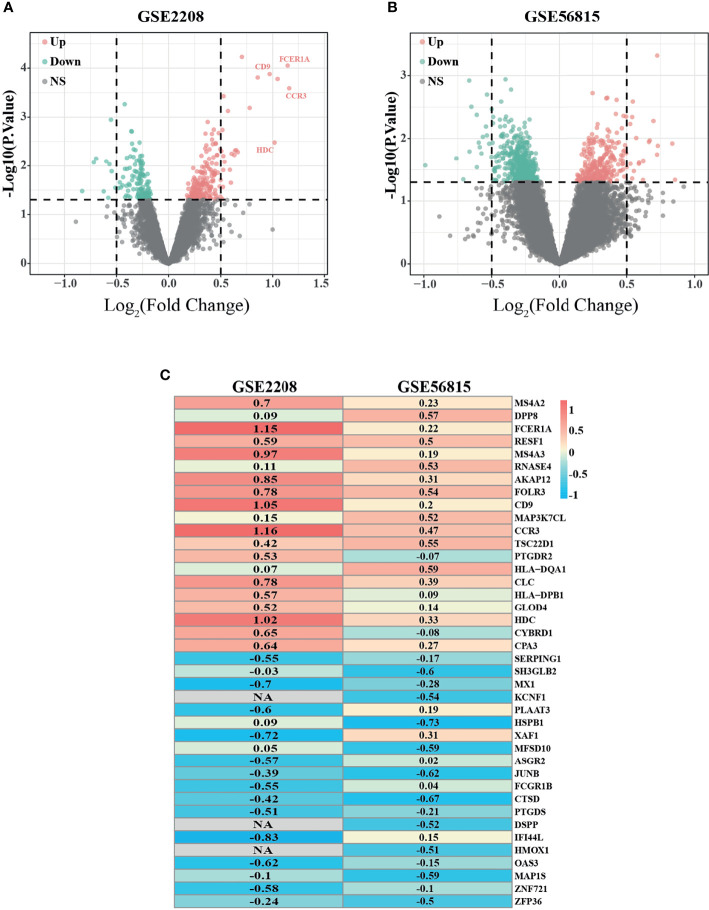
Identification of differentially expressed genes (DEGs) with the cut off criteria of P-Value <0.05. **(A)** Volcano plot of DEGs for GSE2208 datasets. **(B)** Volcano plot of DEGs for GSE56815 datasets. **(C)** The heatmap of top 40 (20 upregulated DEGs and 20 downregulated DEGs) DEGs according to RRA algorithm. As the color goes from red to blue, indicating the changing from up to downregulation. NA represents genes with no statistical significance in GSE2208, but it can be reflected in RRA algorithm because of its high ranking and small P value in GSE56815. Similar results can be referenced in https://doi.org/10.3389/fonc.2019.00996.

According to the previous criteria, samples from two microarray datasets, GSE2208 and GSE56815, were downloaded from the GEO database. The two datasets contain 50 samples in total, one half were from premenopausal high BMD women, the other half were from postmenopausal low BMD women. The details of each dataset are shown in **Table 1**. To explore the most valuable differential genes, we first analyzed the DEGs by using R software through “Limma” package and presented it in the form of Volcano plot (**Figures 1A, B**). Then the “Robust Rank Aggregation” package (RRA) was used to screen Robust genes with P-Value <0.05 and |logFC| >0.5. Finally, 38 DEGs that were downregulated and 30 DEGs that were upregulated were found. The top 20 downregulated DEGs and top 20 upregulated DEGs were presented in a heatmap (**Figure 1C**).

### Functional Enrichment Analysis of Robust DEGs

**Figure 2:**
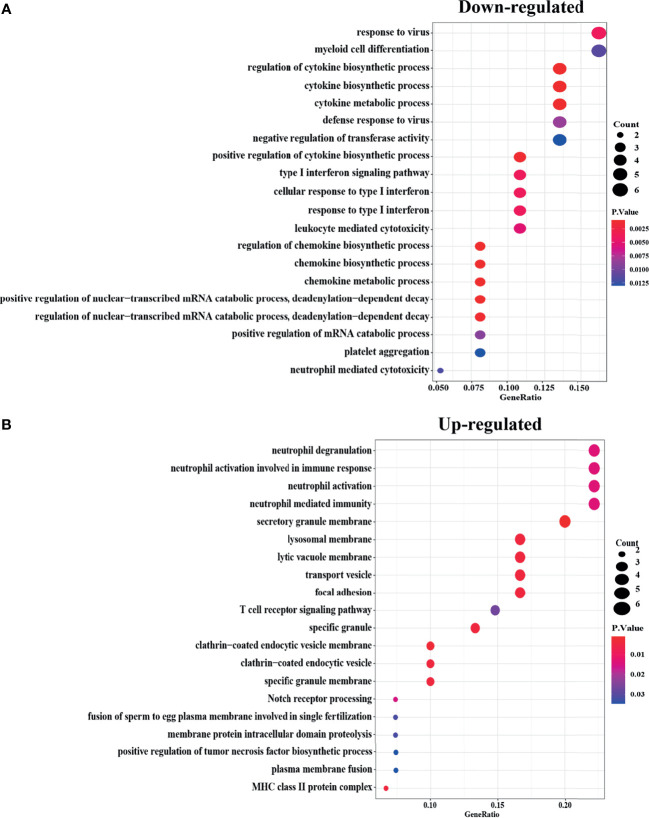
GO enrichment of Robust DEGs with the cut off criteria of P-Value <0.05. The bubble color represents P-Value in enriched pathways, and the size of the bubble represents the gene number. **(A)** Downregulated Robust DEGs in three parts of GO enrichment (BP, CC, and MF). **(B)** Upregulated Robust DEGs in three parts of GO enrichment (BP, CC, and MF). DEG, differentially expressed gene; GO, Gene Ontology; BP, biological process; CC, cellular component; MF, molecular function.

**Figure 3:**
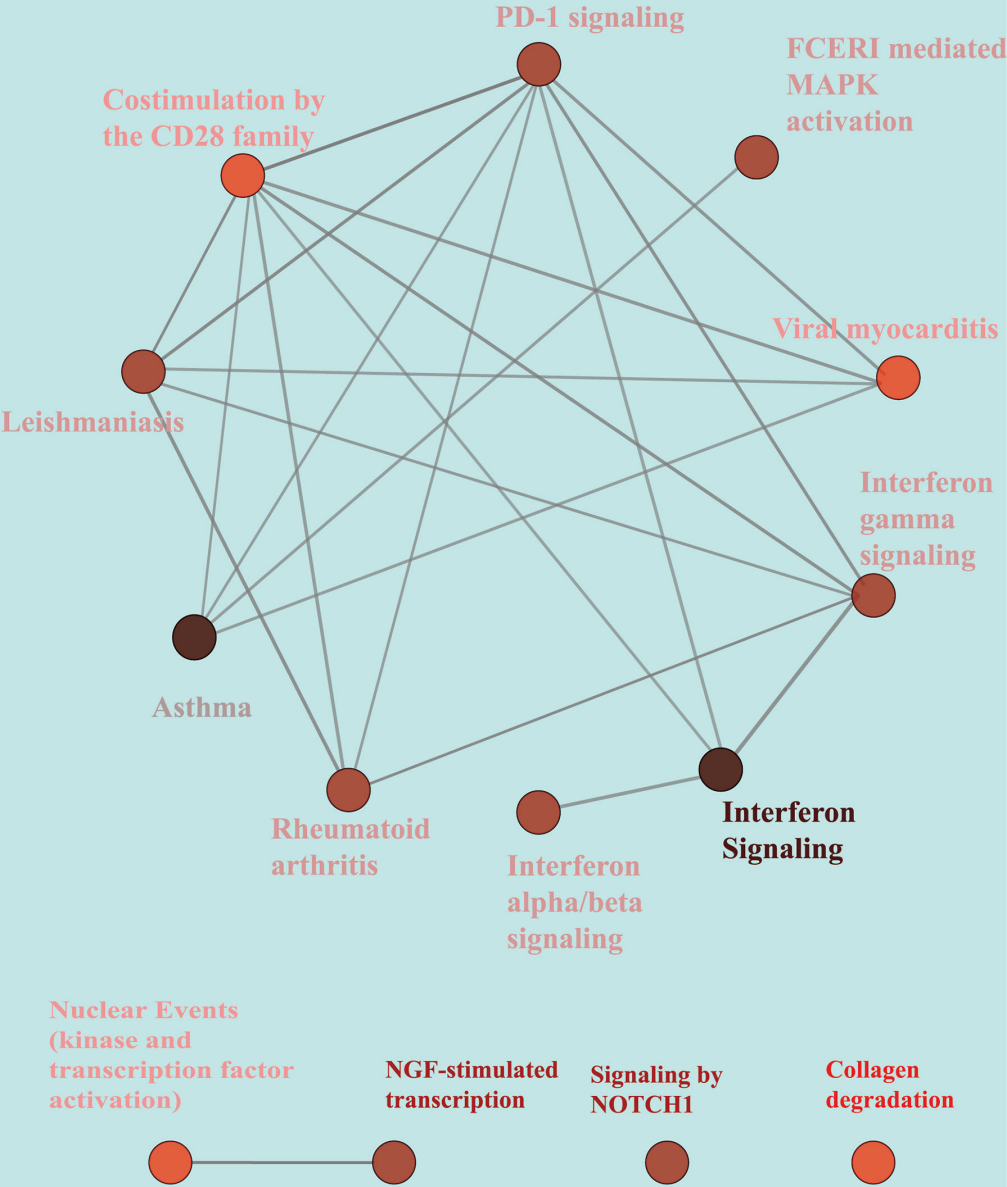
KEGG/Rectome pathways enrichment analysis of Robust DEGs. KEGG, Kyoto Encyclopedia of Genes and Genomes. Rectome, Rectome pathway database.

To figure out the functions of Robust DEGs, “clusterprofiler”, an R package, was used for GO enrichment (biological process, molecular function, and cellular component) (**Figure 2**) and CLUEGO was used to find out the statistically significant pathways through the KEGG/Reactome database (**Figure 3**). To make sure the outcomes are clear and intuitive, the upregulated DEGs and downregulated DEGs were analyzed separately in GO enrichment. The downregulated genes, the results revealed that myeloid cell differentiation which is significantly enriched in GO analysis, are highly associated with the emergence of monocytes and granulocytes. Additionally the complicated biological processes, namely, regulation of cytokine biosynthetic process, cytokine biosynthetic process, and cytokine metabolic process, have all been linked to osteoporosis. In the upregulated genes, immune-related processes, namely, neutrophil degranulation, neutrophil activation involved in immune response, neutrophil activation, neutrophil mediated immunity, and immunoglobulin binding, have long been recognized to have a relationship with the skeletal systems.

There were fourteen pathways significantly enriched in robust DEGs. Notch signaling pathway is of great importance in Human Skeletal Diseases, and collagen degradation, to a large extent, affecting the balance of bone formation and bone resorption. Moreover, the immune system mediated by PD-1 signaling and the activation of MAPK signaling pathway are also involved in bone regulation. The genes contained in each node and the number of genes, and the P-value of the node are detailed in the **Supplementary Material** (**Data Sheet 1**).

### PPI Network Construction and Its Sub-Module Analysis

**Figure 4:**
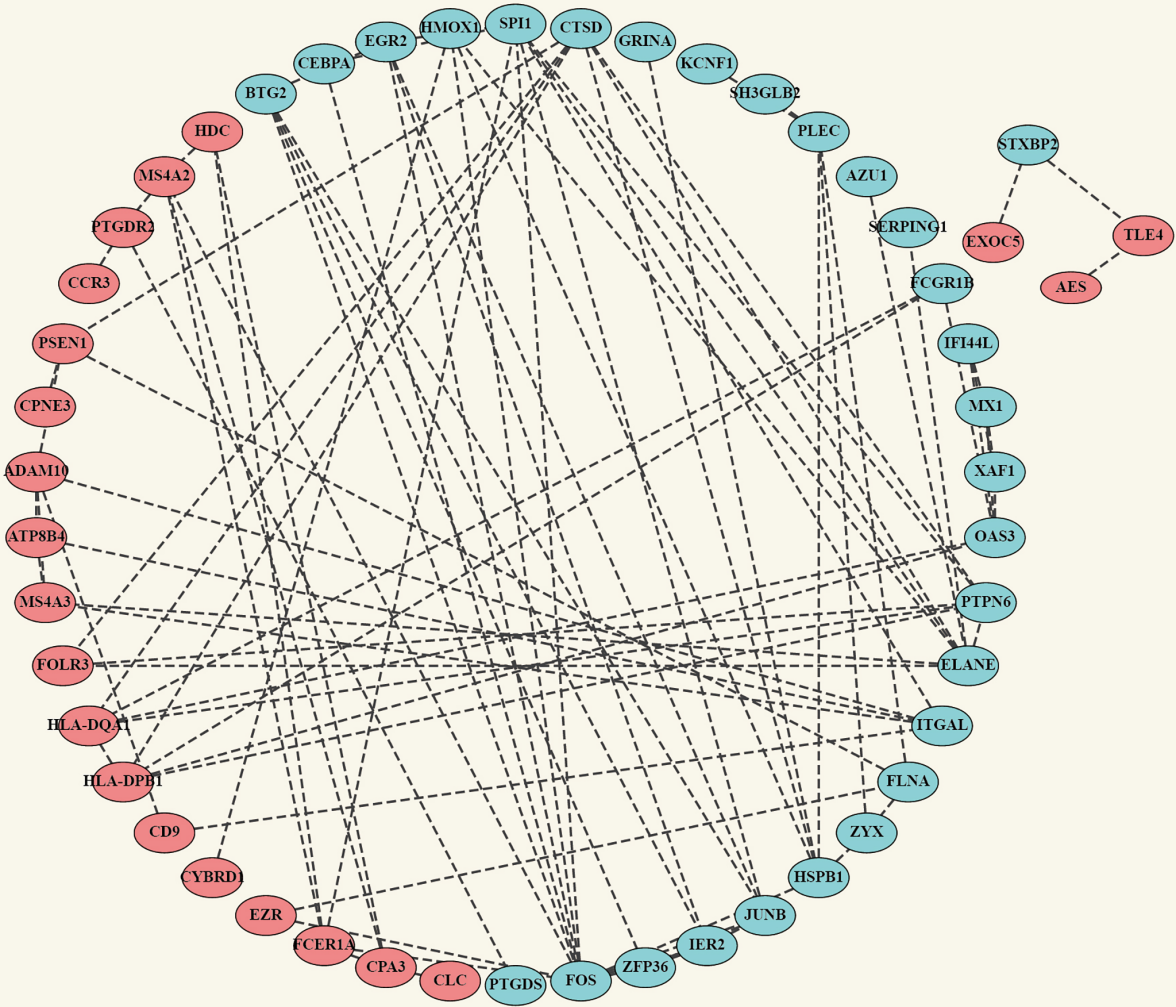
Construction of PPI network. Complete PPI network diagram which contains 66 nodes and 91 edges with the confidence >0.4. Upregulated Robust DEGs are marked in red while Downregulated Robust DEGs are marked in blue. PPI, Protein–Protein Interaction Network.

**Figure 5:**
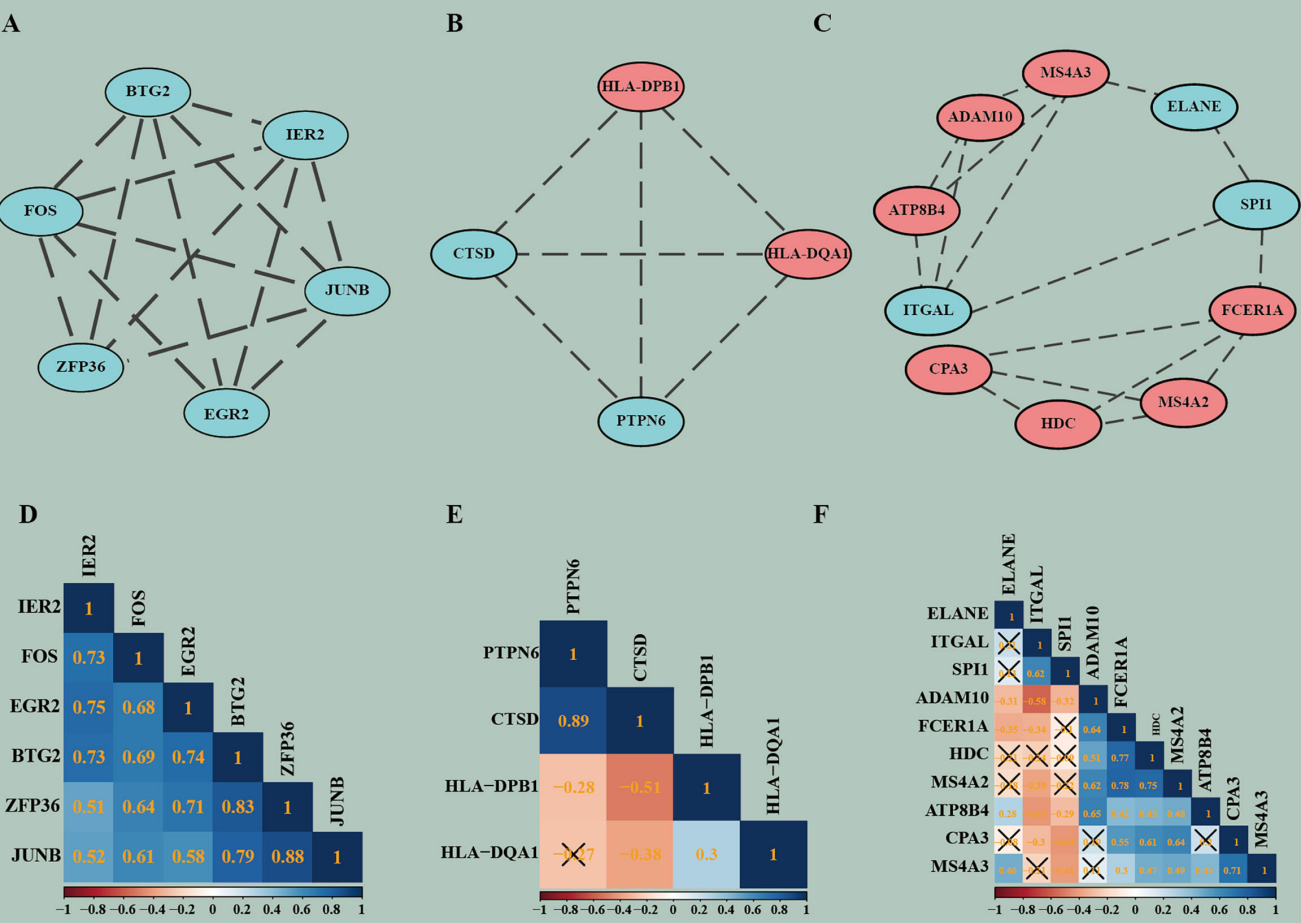
The highly connected modules of PPI and the correlation matrix corresponding to each module. **(A–C)** Modules of PPI network. **(D–F)** Correlation matrixes of highly connected genes in each module. In the modules, red represents genes that are upregulated and blue represents genes that are downregulated. In the correlation analysis, blue indicates positive correlations, red indicates negative correlations, and higher values indicate higher correlations. Squares marked with a cross indicate that the correlation analysis between genes is not statistically significant (P > 0.05).

The robust DEGs were used to construct the PPI network for further study. The results show that with confidence >0.4, a PPI network which contains 66 nodes and 91 edges was constructed (**Figure 4**). MCODE plugin was used to explore those highly connected modules, and three of them were found (**Figures 5A–C**). The genes in Module one were connected with TNF signaling pathway and osteoclast differentiation while genes in Module two were associated with antigen processing, presentation, and antigen receptor-mediated signaling pathway. Besides, the BP of genes in Module three was particularly related to extracellular matrix disassembly.

### Correlation Analysis of Highly Connected Genes in Each Module

Pearson’s correlation coefficient, as an analysis method, and package “correplot” were used to calculate and visualize statistically significant (P-Value <0.05) and highly correlated genes in the modules by correlation analysis. Also, genes that are not statistically different are indicated by a cross. By this method, we identified genes with significant positive correlations, such as PTPN6 vs CTSD and JUNB vs ZFP36. We also found many genes with significant negative correlations, such as CTSD vs HLA-DPB1 and ADAM10 vs ITGAL. These findings provide us new insight for finding potential biomarkers (**Figures 5D–F**).

### Identification of Hub Genes and Their Expression in Different Dataset

**Figure 6:**
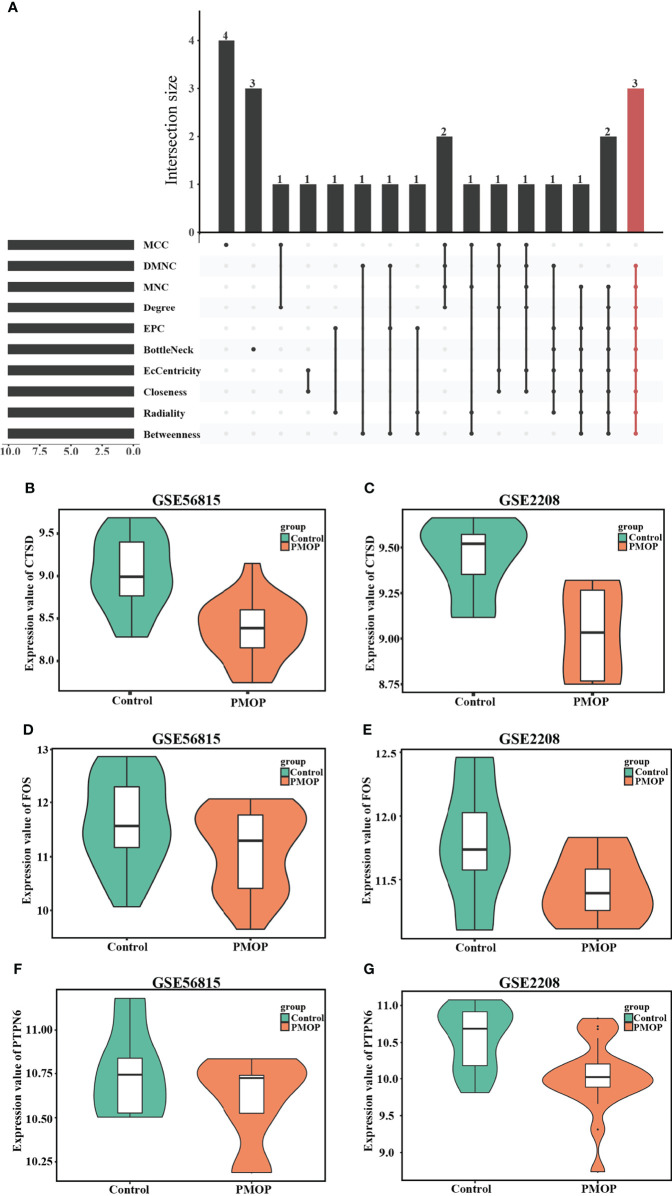
Identification of hub genes and Boxplot of hub genes that expressed consistently across two datasets. **(A)** Hub genes were identified by intersection of 50 genes from 10 algorithms, namely, MCC, DMNC, MNC, Degree, EPC, BottleNeck, EcCentricity, Closeness, Radiality, and Betweenness. The consistent expression of CTSD in **(B)** GSE56815 and **(C)** GSE2208. The consistent expression of FOS in **(D)** GSE56815 and **(E)** GSE2208. The consistent expression of PTPN6 in **(F)** GSE56815 and **(G)** GSE2208.

Cytohubba, as a Cytoscape plugin, is applied to search for the hub genes, and was used to obtain as many of exact hub genes (**Figure 6A**). We picked up the most intersection genes by calculating ten different algorithms, and then we explored the description of those three hub genes, namely, their full names, synonyms and functions (**Table 3**). Subsequently, we presented the consistent expression of these three hub genes of different datasets by violin plots (**Figures 6B–G**).

**Table 3:** Description of the Hub genes.

Gene	Full name	Synonyms	Function
FOS	proto-oncogene c-fos	G0S7	On TGF-beta activation, forms a multimeric SMAD3/SMAD4/JUN/FOS complex at the AP1/SMAD-binding site to regulate TGF-beta-mediated signaling. Has a critical function in regulating the development of cells destined to form and maintain the skeleton. It is thought to have an important role in signal transduction, cell proliferation and differentiation.
CTSD	Cathepsin D	CPSD	Acid protease active in intracellular protein breakdown. Plays a role in APP processing following cleavage and activation by ADAM30 which leads to APP degradation
PTPN6	Tyrosine-protein phosphatase non-receptor type 6	HCP, PTP1C	Modulates signaling by tyrosine phosphorylated cell surface receptors such as KIT and the EGF receptor/EGFR. The SH2 regions may interact with other cellular components to modulate its own phosphatase activity against interacting substrates. Together with MTUS1, induces UBE2V2 expression upon angiotensin II stimulation. Plays a key role in hematopoiesis.

### Validation of the Hub Genes *In Vivo*

**Figure 7:**
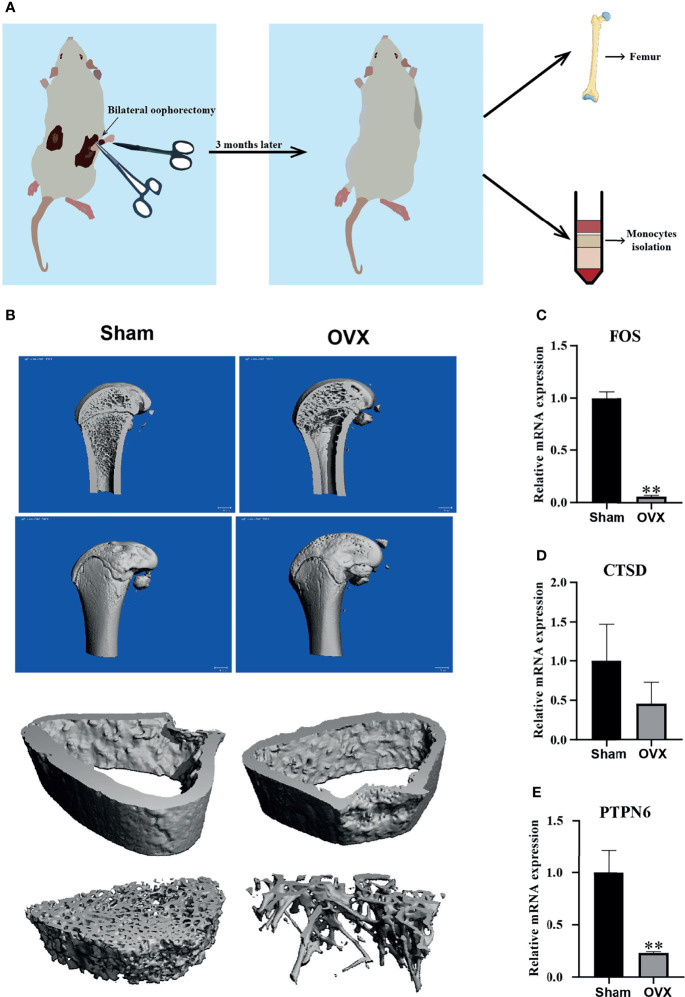
Validation of the hub genes *in vivo*. **(A)** A diagram exhibited how we validate our results of hub genes. **(B)** Representative μ-CT analysis image of SD rat femur in sham and OVX group, and femur 1.5 mm thick below the growth plate was used for analysis of bone cortex and cancellous bone. **(C–E)** The mRNA expression level of three hub genes (FOS, CTSD, and PTPN6) in sham group and OVX group. Compared with the Sham group, **p < 0.01.

In order to verify the hub genes *in vivo*, OVX rats were used to replace the PMOP patients for the validation. A diagram exhibited how we validate our results of hub genes (**Figure 7A**). We confirmed that the OVX model was successfully established through the μ-CT analysis femur of the SD rat three months later after surgery, and the femur 1.5 mm thick below the growth plate was used for analysis of bone cortex and cancellous bone. The μ-CT analysis results showed that the OVX group had fewer trabeculae than the sham group, whereas there was little difference in bone cortex (**Figure 7B**). Meanwhile, the mRNA of monocytes isolated from whole blood was used to detect the expression of those three hub genes (FOS, CTSD, and PTPN6) that we found by bioinformatics analysis, and the real-time PCR results showed that the mRNA expression level of the three hub genes in OVX group was lower than that in the sham group (**Figures 7C–E**). These results are consistent with the bioinformatics analysis results, indicating that the hub genes may be used as biomarkers of PMOP. However, the exact mechanism needs to be further elucidated.

## Discussion

Postmenopausal osteoporosis (PMOP) is a complex bone metabolism disorder characterized by the loss of bone minerals and an increased risk of bone fracture. PMOP is recognized as a metabolism disease which is caused by the imbalance between bone formation and bone resorption after estrogen deficiency. In previous studies, many susceptibility genes for PMOP (35,36), especially cytokines and osteoprotegerin, which are involved in bone remodeling balance, have been identified. It is well known that TNF-α has been shown to affect bone metabolisms by promoting osteoclast differentiation and inhibiting osteoblast differentiation (37). Spon1 may play an important role in the etiology of PMOP (20). However, the use of RRA to detect DEGS in PMOP has not been reported. Therefore, it is very important to investigate the cause of this disease and to find potential treatments by identifying the susceptibility gene of PMOP. This study was the first to search and combine microarrays on PMOP in GEO, in particular, the detection and analysis of blood monocytes, which has not been studied in previous research. It is well known that blood monocytes are precursors of osteoclasts (38), and blood monocytes can secrete a number of potent cytokines important for osteoclast differentiation, activation, and apoptosis (39). The aim of our research was to identify key genes and pathways involved in the pathogenesis of PMOP. Therefore, two microarrays were included in our studies, gene expression profiles of PMOP were compared with controls, and the RRA analysis was adopted to integrate results with more statistical power. Furthermore, PPI network construction and Gene Ontology (GO) enrichment were performed to understand the potential biological function of the DEGs. Meanwhile, hub genes were calculated for further study.

There were 68 DEGs filtered out from two datasets with 38 downregulated genes and 30 upregulated. FOS, PTPN6, and CTSD were the most significant genes among these DEGs, which were identified as hub genes by PPI network analysis. As we all know, FOS is a member of the AP-1 transcription factor complex, which is a central regulator for many physiological functions. In osteoimmunology, Fos/AP-1, as osteoclastogenic transcription factor, plays a critical role in inflammatory bone loss by regulating genes like NFATc1 and also the interferon system (40–42). In the previous study, Sugatani found a positive feedback loop of c-Fos/miR-21/PDCD4, which regulates osteoclastogenesis (43). Besides, the FOS-related protein Fra-2 controls osteoclast size and survival (44).

PTPN6, a cytoplasmic phosphatase, functions to prevent autoimmune and interleukin 1 receptor (IL-1R)-dependent caspase-1-independent inflammatory disease. It has dual function in negative regulation of p38 mitogen-activated protein kinase activation to control tumor necrosis factor and IL-1α/β expression, thereby inhibiting caspase-8-and Ripk3/Mlkl-dependent inflammation (45). Moreover, PTPN6 can regulate the activity of over 50 cytoplasmic signaling proteins and cell surface receptors. It has been reported that PTPN6 mediated autophagy contributes to the inhibition of macrophage foam cell formation by the D3-VDR-PTPN6 axis (46), and participates in the epigenetic regulation mechanism of advanced chronic myeloid leukemia (47). Besides, PTPN6 was upregulated in the coculture system which consists of primary myeloma and healthy donor hematopoietic bone marrow, indicating that PTPN6 plays an important role in microenvironment interactions (48). Another hub gene, CTSD, which is one of the major lysosomal proteases indispensable for the maintenance of cellular proteostasis by turning over substrates of endocytosis, phagocytosis, and autophagy. Consequently, CTSD deficiency leads to a strong impairment of the lysosomal-autophagy machinery (49). However, there were few studies about the definite function and mechanisms of PTPN6 and CTSD in PMOP.

In our GO enrichment analysis, we divided DEGs into two groups. In the downregulated genes, results revealed that myeloid cell differentiation is highly associated with the emergence of monocytes and granulocytes, and the regulation of cytokine metabolic process and cytokine biosynthetic process. These complicated biological processes have all been linked to osteoporosis (11,50,51). While in the upregulated genes, immune-related processes such as neutrophil activation, neutrophil degranulation involved in immune response, neutrophil activation, neutrophil mediated immunity and immunoglobulin binding have long been recognized to have a relationship with the skeletal systems (52).

In the current study, we established a postmenopausal osteoporosis model in rats by bilateral oophorectomy. We found that PTPN6, CTSD, and FOS from blood monocytes in the OVX group were lower than that in the control group, which is consistent with our bioinformatic analysis results. However, monocytes isolated from the whole blood of older women may yield different sequencing data than OVX rats. It is a pity that we cannot get sequencing data from monocytes of humans because of strict social rules and ethical requirements during the COVID-19 pandemic. These findings provide a new target for further research on the role of blood monocytes in the development of postmenopausal osteoporosis. However, the occurrence of postmenopausal osteoporosis involves estrogen level, immune factors, osteoblast activity, and many other factors. It will be worthwhile to further study the roles of PTPN6, CTSD, and FOS in other cells or biological processes except osteoclasts, and explore their significance in the occurrence and development of osteoporosis from the perspective of proteomics and protein modification omics. At last, we believe our results from the GEO data and animal experiment validation may provide a fresh light into the role of monocytes in premenopausal osteoporosis and identified FOS, PTPN6, and CTSD as potential biomarkers for premenopausal osteoporosis.

In conclusion, by using the RRA approach, our study successfully provided a deeper understanding of the overall molecular changes in the pathogenesis of PMOP and identified several potential therapeutic candidates, namely, FOS, PNPT6, and CTSD, as central genes. Besides, through GO and KEGG pathway analysis, we found that these differential genes were mainly enriched in cytokine metabolic process and cytokine biosynthetic process, and may be involved in Notch signaling pathway and immune system. Collectively, this study may provide reliable molecular biomarkers for diagnosis, screening and prognosis of PMOP. It also lays a foundation for exploring new therapeutic targets of PMOP. However, the underlying molecular mechanisms have not yet been fully elucidated. In the future, more experiments are needed to verify the changes of gene expression and it is necessary to collect a large number of bone marrow tissues from patients with PMOP and bone tissues from normal controls for further research.

## Data Availability Statement

The datasets presented in this study can be found in online repositories. The names of the repository/repositories and accession number(s) can be found in the article/**Supplementary Material**.

## Ethics Statement

The animal study was reviewed and approved by The Ethics Committee of Chongqing Medical University.

## Author Contributions

Funding acquisition, YD and LC. Investigation, H-JC, J-HJ, Y-YY, and H-HL. Software, Y-RD. Supervision, CH. Validation, Y-XD and W-GH. Writing—Original draft, Y-XD and W-GH.

## Funding

This work was supported by the National Natural Science Foundation of China (NSFC, 81572226 to B-CH) and the youth project (Grant No. 82000836 to YD).

## Conflict of Interest

The authors declare that the research was conducted in the absence of any commercial or financial relationships that could be construed as a potential conflict of interest.

## Publisher’s Note

All claims expressed in this article are solely those of the authors and do not necessarily represent those of their affiliated organizations, or those of the publisher, the editors and the reviewers. Any product that may be evaluated in this article, or claim that may be made by its manufacturer, is not guaranteed or endorsed by the publisher.
